# Clinical characteristics and risk factors for mortality among COVID-19 hospitalized patients in UAE: Does ethnic origin have an impact

**DOI:** 10.1371/journal.pone.0264547

**Published:** 2022-03-02

**Authors:** Salah AbuRuz, Ahmad Al-Azayzih, Sham ZainAlAbdin, Rami Beiram, Mohammed Al Hajjar

**Affiliations:** 1 Department of Pharmacology and Therapeutics, College of Medicine and Health Sciences, The United Arab Emirates University, Al Ain, United Arab Emirates; 2 Department of Clinical Pharmacy, Faculty of Pharmacy, The University of Jordan, Amman, Jordan; 3 Department of Clinical Pharmacy, Faculty of Pharmacy, Jordan University of Science and Technology, Irbid, Jordan; 4 Department of Pharmacy, Al Ain Hospital, Al Ain, United Arab Emirates; University Hospital of Modena (Italy), Respiratory Diseases Unit, ITALY

## Abstract

**Background:**

The relationship between COVID-19 patient’s clinical characteristics and disease manifestation remains incompletely understood. The impact of ethnicity on mortality of patients with COVID-19 infection is poorly addressed in the literature. Emerging evidence suggests that many risk factors are related to symptoms severity and mortality risk, emphasizing the necessity of fulfilling this knowledge gap that may help reducing mortality from COVID-19 infections through tackling the risk factors.

**Aims:**

To explore epidemiological and demographic characteristics of hospitalized COVID-19 patients from different ethnic origins living in the UAE, compare them to findings reported across the globe and determine the impact of these characteristics and ethnicity on mortality during hospitalization.

**Methods:**

A single center, retrospective chart review study of hospitalized COVID-19 patients was conducted in a large COVID-19 referral hospital in UAE. The following outcomes were assessed: patients’ clinical characteristics, disease symptoms and severity, and association of ethnicity and other risk factors on 30-day in hospital mortality.

**Results:**

*A total of* 3296 patients were recruited in this study with an average age of 44.3±13.4 years old. Preliminary data analysis indicated that 78.3% (n = 2582) of cases were considered mild. Average duration of hospital stay was 6.0±7.3 days and 4.3% (n = 143) were admitted to ICU. The most frequently reported symptoms were cough (32.6%, n = 1075) and fever (22.2%, n = 731). The 30-day mortality rate during hospitalization was 2.7% (n = 90). Many risk factors were associated with mortality during hospitalization including: age, respiratory rate (RR), creatinine, and C-reactive protein, oxygen saturation (SaO_2_), hemoglobin, hematocrit, ferritin, creatinine, C-reactive protein, anemia, COPD, Chronic kidney disease, dyslipidemia, Vitamin-D Deficiency, and ethnic origin (p <0.05). Multiple logistic regression analysis showed that higher mortality rates during hospitalization was associated with anemia, chronic obstructive pulmonary disease (COPD), chronic kidney disease, and Middle Eastern origin (p<0.05).

**Conclusion:**

The results indicated that most COVID-19 cases were mild and morality rate was low compared to worldwide reported mortality. Mortality rate during hospitalization was higher in patients from Middle East origin with preexisting comorbidities especially anemia, COPD, and chronic kidney disease. Due to the relatively small number of mortality cases, other identified risk factors from univariate analysis such as age, respiratory rate, and Vitamin-D (VitD) deficiency should also be taken into consideration. It is crucial to stratify patients on admission based on these risk factors to help decide intensity and type of treatment which, possibly, will reduce the risk of death.

## Introduction

Since the early 2020, Corona virus (SARS-CoV-2) has rapidly spread over the world causing a communicable respiratory infection known as “COVID-19”, with symptoms varying from person to person. Up to April 2021, 8,609,860 cases were confirmed with consequent 173,334 disease- related deaths in the Eastern Mediterranean region (including the Middle East). Despite the diversity of healthcare systems in the countries of Eastern Mediterranean region, their restriction of social, educational, and economic movements has limited the enormous prevalence of the infection [1].

In the UAE, the virus was first detected in the early March 2020. In January 2021, a total of 370,425 cases were registered with 1,125 reported disease- related deaths. Although UAE is the central hub of national diversity with multi-ethnicity and cultural concepts, these numbers were much lower than numbers reported by many other countries due early implementation of governmental efforts in combating this crisis; including social distancing, lockdown, sterilization campaigns, restricted regulations in the tourism and entertainment destinations, cases isolation, and mandatory masking in all public places [2, 3].

Researchers have devoted their efforts to identify the major risk factors of COVID-19- related death and negative health outcomes in different populations. According to previously published records, age, metabolic syndrome, cardiovascular disease, oxygen saturation as well as chronic obstructive pulmonary disease (COPD) were the most common risk factors of COVID-19- related death and severe illness [4–6].

Although majority of infected patients had mild to moderate symptoms including cough, fever and fatigue, minority of them were vulnerable to have severe illness as they were either admitted to the intensive care unit (ICU) or eventually died [7]. Acute respiratory distress is a common severe symptom followed COVID-19 among patients suffering from the abovementioned co-morbidities and complications. Most of these cases were admitted to the ICU (40.0–96.0%) and the need for an invasive mechanical ventilator (IMV) was variable between patient and another. However, the use of IMV was invariably associated with higher death rate exceeding 16.0% [8–10].

Moreover, the overwhelming burden of this crisis has been associated with high pressure on the capacity of the healthcare systems, which in turn negatively affects the healthcare workers [11]. Therefore, it is crucial to stratify patients according to the severity of the disease and risk of morbidity and mortality. This can be achieved by systematic analysis of the hospitalized cases by identifying their clinical data, symptoms, and chronic illnesses followed by comprehensive evidence- based clinical decisions to manage severe cases and those at risk of death and save patients’ lives.

The aims of this study were to explore epidemiological characteristics of hospitalized COVID-19 patients from different ethnic origins living in the UAE, and to determine the impact of demographic, clinical characteristics and ethnicity on mortality rate during hospitalization.

## Materials and methods

### Study design and participants

This is a retrospective chart review of patients with confirmed COVID-19 who were above 18 years old and admitted to a large government tertiary care centre in Al-Ain city at the UAE during the period from March to June 2020, the period when the pandemic peaked in the UAE. Infection was confirmed with the polymerase-chain reaction (PCR) [12]. All adult patients diagnosed with COVID-19 during the study period were eligible for inclusion. The study site is the largest in the region and is one of the main COVID-19 referral centres in UAE. During the study period, it was the sole designated facility in the city of Al Ain for COVID-19 patients of all ages The hospital capacity is 415 beds, including 85 negative-pressure intensive care beds. All confirmed/suspected cases of COVID-19, presenting either to any public or private health facility in the city throughout the study period, were transferred to this hospital, it was also receiving patients from all over UAE.

The following were the exclusion criteria

1. Pediatrics (Age <18 years old)
2. Pregnant patients
3. Patients admitted for Surgery
4. Patients with Co-infection with other diseases

### Definitions and diagnosis

The diagnosis of SARS-Cov2 was established as per the World Health Organization (WHO), using nasopharyngeal real-time polymerase chain reaction (RT PCR). All comorbidities were identified from the electronic medical record. These include only physician confirmed diagnosis. These comorbidities include diabetes, hypertension, anemia, vitamin D deficiency, dyslipidemia, chronic kidney disease, asthma, Cancer, and COPD. Disease severity was classified according to the WHO guidelines for classifying COVID-19 cases into mild, moderate and severe. Mild cases were identified by having cough and/or fever with other symptoms including nausea, vomiting, diarrhoea, headache and loss of smell with reported shortness of breath/ dyspnoea. Moderate cases have same as mild cases symptoms and show evidence of lower respiratory disease on the imagining systems and shortness of breath/ dyspnoea with oxygen saturation of ≥94.0%. Severe cases were associated with oxygen saturation lower than 94.0% [13].

Patients were followed up until discharge or death. Clinical recovery was defined as hospital discharge based on the absence of clinical symptoms requiring inpatient.

### Data collection

Data were collected from the electronic medical records from March to May 2020. An IT engineer retrieved all the patients’ data from the database in Excel sheet. Two of the research investigators revised, cleaned, and coded the data independently to check for inter-rater reliability. Any disagreement was discussed between the two investigators.

The following data were collected: demographic and clinical characteristics, vital signs on admission, co-morbidities, symptoms, and COVID-19 severity, need for oxygen supply or ventilation, and duration of hospitalization. Results of Laboratory tests were also collected, including complete blood count (CBC), creatinine, liver enzymes, ferritin, and HbA1CThe 30-days in hospital mortality rate was also collected.

### Ethical consideration

This study was approved by Covid-19 IRB committee at the Department of Health in the United Arab Emirates (reference number: DOH/CVDC/2020/1121, 3^rd^ of June 2020). Since it is a retrospective observational study of patients’ records, the need for an informed consent form was waived. Patients’ identity was kept anonymous, and all patients were identified by their medical record number.

### Statistical analysis

Data extracted from patients’ medical records was analyzed using the Statistical Package of Social Sciences version 22 (IBM SPSS Statistics for Windows, Version 24.0. Armonk, NY: IBM Corp.). Categorical variables were represented as frequencies (percentages and 95.0% CI), whereas mean± standard deviation (SD) was calculated for continuous variables.

#### Outcome assessment

To explore the risk factors associated with in-hospital mortality, univariate statistics and multivariate logistic regression analysis were used. Demographic and clinical risk factors of death due to COVID-19 infection were determined using chi-squared tests for categorical variables and Student’s t-test or Mann-Whitney’s U for continuous variables depending on variable distribution. Multivariate logistic regression was used to identify the independent risk factors of death upon COVID-19 infection. To account for potential confounding effect, factors that were found significant using univariate analysis were entered into logistic regression.

For logistic regression, entry was set at 0.05 and removal at 0.0.05 using backward Wald. Odds ratios (OR) and adjusted OR (aOR) with 95% confidence interval (CI) were also estimated. Checking for collinearity was carried out using tolerance and Variance Inflation Factor. A p-value of <0.05 was considered statistically significant.

## Results

### Demographics and clinical data

During the study period, we identified 3492 unique admissions with a Covid-19 diagnosis. Of those admissions, 3296 (94.4) admissions fulfilled the inclusion criteria.

Table 1 represents the demographic and clinical characteristics of the study patients. Majority of patients were males (76.3%, n = 2515) with an average age of 44.3 ± 13.4 years old. Most of the patients were smokers or have a history of smoking (76.4%, n = 2519). The average duration of hospitalization was 6.0 ± 7.2 days and 4.3% (n = 143) were admitted to the ICU. The mortality rate during hospitalization was 2.7% (n = 90). Most of deaths occurred within the first 24 days (87 patients). Table 2 shows symptoms, severity of the infection in addition to the associated medical conditions. Cough was the most frequently reported symptoms for COVID-19 (32.6%, n = 1075), followed by fever (22.2%, n = 731). Loss of smell or taste was rarely reported by the patients (1.1%, n = 35). Majority of the cases were mild (78.3%, n = 2582) and only 6.7% (n = 220) were severe with oxygen saturation level <94.0%. The most diagnosed health problems among the studied patients were pneumonia (70.0%, n = 2307), hypertension (28.6%, n = 944) and diabetes mellitus (27.4%, n = 903). Further clinical details are illustrated in the Tables 1 and 2.

**Table 1:** Demographics and clinical characteristics of the patients (N = 3296).

***Variables***	***N (%)***
Gender	
*Males*	2515 (76.3)
*Females*	781 (23.7)
Smoking	
*Non-smokers*	777 (23.6)
*Current smokers*	2475 (75.1)
*Former smokers*	44 (1.3)
Ethnic Origin	
*Emirati*	486 (14.7)
*Other Middle Eastern (mainly Syrian and Jordanian)*	289 (8.8)
*Asians (Mainly India*, *Pakistan*, *Bangladesh*, *Philippines)*	2196 (66.6)
*Africans*	259 (7.9)
*Other minorities (manly western)*	66 (2.0)
ICU admission	143 (4.3)
Death	90 (2.7)
***Variables***	***Mean ± SD***
Age	44.3 ± 13.4
HR (beats/min)	92.5 ± 15.6
RR (breaths/min)	19.8 ± 5.1
SBP (mmHg)	134.4 ± 19.1
DBP (mmHg)	79.7 ± 15.2
SaO_2_ (%)	97.7 ± 3.64
BMI (Kg/m^2^)	27.4 ± 8.2
HBA1c (%)	8.5 ± 2.3
D-dimer (mg/L)	0.9 ± 2.6
Haemoglobin (g/L)	137.7 ± 18.7
Haematocrit (L/L)	0.41 ± 0.05
Total Bilirubin (micromole/L)	8.9 ± 6.0
C- Reactive Protein (mg/L)	35.4 ± 56.4
Creatinine (micromole/L)	83.8 ± 63.5
Ferritin Level (mcg/L)	339.6 ± 247.5
INR	1.1 ± 0.6
Potassium level (mmol/L)	4.0 ± 0.5
Sodium level (mmol/L)	138.2 ± 3.8
Liver Test *(IU/L)*	
*Alkaline Phosphatase*	81.3 ± 39.4
*AST*	35.3 ± 27.0
*ALT*	37.1 ± 33.2
Duration of hospitalization	6.0 ± 7.2

HR: heart rate, RR: respiratory rate, SBP: systolic blood pressure, DBP: diastolic blood pressure, SaO2: saturated oxygen, BMI: body mass index, HbA1C: haemoglobin A1c, INR: international normalised ratio, ALT: alanine transaminase, AST: aspartate aminotransferase.

**Table 2:** Symptoms, severity of infection, and associated comorbidities among the patients (n = 3296).

***Symptoms***	***N (%)***
Cough	1075 (32.6)
Shortness of breath/Dyspnea	568 (17.2)
Fever	731 (22.2)
Sore throat	241 (7.3)
Body/muscle pain/malaise	536 (16.3)
Nausea/Vomiting/Diarrhoea	182 (5.5)
Loss of smell/taste	35 (1.1)
Headache	125 (3.8)
***Severity***	***N (%)***
Mild	2582 (78.3)
Moderate	493 (15.0)
Severe	221 (6.7)
***Co-morbidities***	***N (%)***
Hypertension	944 (28.6)
Diabetes Mellitus	903 (27.4)
Dyslipidaemia	506 (15.4)
Myocardial Infraction	30 (0.9)
Asthma	165 (5.0)
Anaemia	377 (11.4)
Cancer	80 (2.4)
Chronic Obstructive Pulmonary Disease (COPD)	36 (1.1)
Pneumonia	2307 (70.0)
Allergic Rhinitis	49 (1.5)
Vitamin-D Deficiency (< 12 ng/mL (<30 nM))	115 (3.5)
Chronic Kidney disease	328 (10.0)

### Risk factors for mortality: Vital signs and laboratory data

Higher RR (p = 0.035), creatinine (p = 0.013) and C- reactive protein (p = 0.026), SaO^2^ (p = 0.023), hemoglobin (p<0.001), hematocrit (p = 0.003) and ferritin (p = 0.024) were significantly associated with Mortality (Table 3).

**Table 3:** Impact of vital signs, age and laboratory parameters on mortality following COVID-19 infection.

*Variable*	*Survivals (Mean± SD)* *(N = 3206)*	*Non survivals (Mean± SD)* *(N = 90)*	*P- Value*
HR	92.5 ± 15.6	92.2 ± 14.4	0.879
RR	19.8 ± 5.0	21.2 ± 6.1	0.035*
SBP	134.6 ± 19.0	131.2 ±23.0	0.096
DBP	81.0 ± 12.3	78.6 ± 13.6	0.067
SaO_2_	97.8 ± 3.6	96.6 ± 5.0	0.023*
BMI	27.4 ± 8.5	28.0 ± 10.4	0.541
HbA1C	8.5 ± 2.2	9.1 ± 3.2	0.436
D-dimer	0.9 ± 2.5	1.1 ± 1.5	0.490
Haemoglobin	137.8 ± 18.6	131.1 ± 22.0	0.001*
Haematocrit	0.4 ± 0.05	0.39 ± 0.06	0.003*
Total Bilirubin	8.8 ± 5.9	8.8 ± 6.3	0.935
C- Reactive Protein	35.0 ± 56.1	50.7 ± 63.9	0.026*
Creatinine	82.7 ± 58.7	124.2 ± 151.9	0.013*
Ferritin Level	337.7 ± 247.0	406.7 ± 255.0	0.024*
INR	1.1 ± 0.6	1.1 ± 0.2	0.829
Potassium level	4.0 ± 0.5	4.1 ± 0.6	0.713
Sodium level	138.3 ± 3.7	138.7 ± 4.6	0.327
Liver Test			
*Alkaline Phosphatase*	80.1 ± 37.0	82.2 ± 31.5	0.660
*AST*	34.8 ± 26.5	38.4 ± 28.2	0.532
*ALT*	37.0 ± 33.2	34.5 ± 27.4	0.259
Duration of hospitalization	5.9 ± 7.1	7.4 ± 8.9	0.066

HR: heart rate, RR: respiratory rate, SBP: systolic blood pressure, DBP: diastolic blood pressure, SaO_2_: saturated oxygen, BMI: body mass index, HbA1C: haemoglobin A1c, INR: international normalised ratio, ALT: alanine transaminase, AST: aspartate aminotransferase.

### Risk factors for mortality: Comorbidities, age, gender and ethnic origin

Table 4 represents the association of co-morbidities with mortality. Dyslipidemia (p< 0.001), anemia (p <0.001), vitamin D deficiency (p = 0.004) and chronic kidney disease (p <0.001) were associated with more than two folds (OR>2) increase in the risk of mortality. Furthermore, COPD showed higher risk of death of 6 times more compared to those with no COPD (p< 0.001). Older age (>65) was also associated with 3 times the risk of death compared with those less than 65 years old. The average age of those who dies was 49.6 compared with 44.2 for survivals (P<0.001).

**Table 4:** Impact of co-morbidities, age, gender and ethnic origin on mortality following COVID-19 infection.

Co-morbidity/Condition	Death rate if there is no disease % (n/N)	Death rate if there is a disease % (n/N)	Odds Ratio (OR) No disease/Disease	CI	P- Value
**Hypertension**	2.5 (58/2352)	3.4 (32/944)	1.39	0.90–2.15	0.090
**Diabetes Mellitus**	2.6 (62/2393)	3.1 (28/903)	1.20	0.77–1.90	0.246
**Dyslipidaemia**	2.3 (64/2789)	5.3 (27/506)	2.44	1.54–3.87	**0.001** *
**Myocardial Infraction**	2.7 (88/3266)	6.7 (2/30)	2.58	0.61–10.99	0.197
**Asthma**	2.7 (84/3131)	3.6 (6/165)	1.37	0.59–3.18	0.295
**Anaemia**	2.3 (68/2918)	5.8 (22/377)	2.60	1.58–4.25	**0.001** *
**Cancer**	2.7 (88/3216)	2.5 (2/80)	0.91	0.22–3.77	0.626
**Chronic Obstructive Pulmonary Disease (COPD)**	2.6 (85/3259)	13.9 (5/36)	6.02	2.29–15.87	**0.001** *
**Pneumonia**	2.7 (27/990)	2.7 (63/2306)	1.00	0.63–1.58	0.551
**Allergic Rhinitis**	2.7 (89/3247)	2.0 (1/49)	0.74	0.10–5.41	0.611
**Vitamin-D Deficiency**	2.5 (81/3180)	7.8 (9/115)	2.83	1.33–5.99	**0.004** *
**Chronic kidney disease**	2.3 (69/2967)	6.4 (21/328)	2.87	1.74–4.75	**0.001** *
**Gender**	Death rate if the gender is female % (n/N)	Death rate if the gender is male % (n/N)	Odds Ratio (OR) female/male	0.76–1.93	0.246
	2.9 (24/832)	2.5 (66/2643)	1.21		
**Ethnic Origin**	Death rate if other origins % (n/N)	Death rate if Middle Eastern % (n/N)	Odds Ratio (OR) Others/Middle eastern	1.31–3.12	**0.002** *
	2.2 (56/2521)	4.4 (34/774)	2.02		
**Age**	Death rate if age >65% (n/N)	Death rate if age < 65% (n/N)	Odds Ratio (OR)	1.85–5.65	**0.001** *
	7.4 (16/217)	2.4 (74/3078)	3.23		

*Significance at p <0.05.

An interesting fasting was that Ethnic Origin was associated with mortality, where Middle Eastern showed almost twice the death rate compared to other origins living in the UAE (OR: 2.02, p = 0.002).

### Risk factors for mortality: Symptoms and severity of the infection

None of the symptoms had a significant impact on the death rate. The risk of death among the mild cases was reduced by 43.0% compared to moderate and severe cases (p = 0.012). On the other hand, severe cases showed almost 2.5 times more risk of death when compared to mild and moderate cases (p = 0.006) (Table 5).

**Table 5:** Impact of COVID-19 symptoms on mortality following COVID-19 infection.

**Symptoms**	**Death rate if there is no symptom % (n/N)**	**Death rate if there is a symptom % (n/N)**	**Odds Ratio (OR) No symptom/ symptom**	**CI**	**P- Value**
**Cough**	2.6 (57/2222)	3.1 (33/1074)	1.20	0.78–1.86	0.233
**Shortness of breath/Dyspnoea**	2.6 (70/2729)	3.5 (20/567)	1.39	0.84–2.30	0.129
**Fever**	2.5 (64/2565)	3.6 (26/731)	1.44	0.91–2.29	0.080
**Sore throat**	2.8 (85/3055)	2.1 (5/241)	0.74	0.30–1.84	0.345
**Body/muscle pain/malaise**	2.6 (71/2760)	3.5 (19/536)	1.39	0.83–2.33	0.133
**Nausea/Vomiting/Diarrhoea**	2.7 (83/3114)	3.8 (7/182)	1.46	0.67–3.21	0.227
**Loss of smell/taste**	2.8 (90/3261)	0.0	0.97	0.97–0.98	0.377
**Headache**	2.8 (89/3171)	0.8 (1/125)	0.29	0.04–2.02	0.136
**ICU Admission**	2.6 (83/3153)	4.9 (7/143)	1.90	0.86–4.20	0.094
**Severity**	**Death rate if different severity N (%)**	**Death rate if same severity N (%)**	**Odds Ratio (OR) Different severity/same severity**	**CI**	**P- Value**
**Mild**	4.1 (29/713)	2.4 (61/2583)	0.57	0.36–0.90	**0.012**
**Moderate**	2.6 (74/2803)	3.2 (16/493)	1.24	0.71–2.14	0.264
**Severe**	2.5 (77/3076)	5.9 (13/220)	2.45	1.34–4.48	**0.006**

## Multivariate logistic regression of risk for mortality

With consideration of the relatively small number of non-survival in the study population and the large number of variables that were significantly associated with mortality, it was not statistically possible to introduce all these significant variables into the logistic regression analysis. The general rule of thumb for logistic regression is to limit the number of independent variables to “10 events (death in this case) per predictor” This means the maximum number of variables should be 9 in this study (90/10). Therefore, we carried out logistic regression in two stages. For stage one, two independent preliminary logistic regression analyses were carried out; one regression was carried for demographic and laboratory data and one for comorbidities and symptoms (only significant variables from univariate analysis were included) in order to identify the most important predictors. After the two preliminary logistic regression analyses, the following eight variables were retained as significant variables: creatinine level, hemoglobin level, ferritin level, age, anemia, chronic kidney disease, COPD, and ethnic origin. Creatinine level was not considered for further analysis as it represents similar construct as chronic kidney disease. Hemoglobin and ferritin levels were not also considered for further analysis as they represent similar construct as anemia. For stage two, those retained variables from stage one, were entered into a final regression analysis (5 variables).

Table 6 illustrates the results of the final stage of logistic regression analysis. Logistics regression analysis demonstrated that anemia, COPD, ethnic origin and chronic kidney diseases were independently associated with greater risk of mortality during hospitalization among patients with COVID-19.

**Table 6:** Multivariate logistic regression analysis of death due to COVID-19 infection.

Variables	Adjusted OR (95.0% CI)	P- Value
**Anemia**	1.76 (1.03–3.00)	0.040
**COPD**	3.06 (1.10–8.53)	0.032
**Chronic kidney disease**	1.86 (1.10–3.26)	0.031
**Ethnic Origin**	1.98 (1.25–3.13)	0.004

## Discussion

This study is the first to present risk factors of death among hospitalized patients with COVID-19 in UAE and explore the influence of multi-ethnic groups living in the UAE on the COVID-19 related mortality. The present study discusses the major predictors of death following COVID-19 among 3296 hospitalized patients in a large governmental hospital at Al-Ain, UAE. Majority of patients who died during hospitalization were previously diagnosed with comorbidities as hypertension, diabetes mellitus, dyslipidemia, or COPD as well as suffering from preexisting health problems including anemia, vitamin D deficiency or kidney problem. This comes in line with previous records in the literature, where comorbidities were associated with higher mortality rates and greater risk of negative health outcomes [14–16]. In the current study, the reported death rate was 2.7%, which is more than the reported death rate in the whole UAE (0.29%) [3]. This can be justified as the study site was one of the main referral site for more severe cases in UAE. This is in addition to receiving all patients from Al-Ain city.

Consistent with previous studies, findings of this study showed that advanced age was associated with higher mortality rate [17]. This can be justified as older adults are more vulnerable to develop more severe symptoms and more likely to die with pre-existing medical problems and comorbidities. In addition, frequent use of antibiotics among adults contributes to superinfection, which makes the condition worse and ends with death [14].

Furthermore, vital signs including higher RR (21.2 ± 6.0%) and lower SaO_2_ (96.7 ± 5.0%) were significantly associated with higher death rates in the present study. Although the prognostic value of dyspnea and hypoxemia, regardless any pre-existing respiratory problem, was not sufficiently emphasized in the literature. However, some previous studies stated that clinical manifestation of shortness of breath/ dyspnea and SaO_2_ of less than 94.0% were markedly prevalent in mortal COVID-19 cases and considered as invariable leading causes of death despite a critical intensive care [13, 17, 18]. Therefore, it is pivotal to analyze the outcomes data from readily measured clinical parameters, especially cases that require triage and critical assessment.

In the current study, COPD was a major independent risk factors of death (almost 6 times more) compared to healthy cases, which supports findings of previous studies [19, 20]. Additionally, dyslipidemia, anemia, vitamin D deficiency and kidney problems were associated with more than two times the risk of death following COVID-19 infection. A recent meta-analysis was consistent with our findings and reported that Vitamin D deficiency increases the risk of death even 15-folds more [21]. Low vitamin D levels are associated with higher levels of inflammatory cytokines, which significantly increases the virulence of the virus causing severe respiratory infection as well as increases the number of thrombotic episodes [22]. Furthermore, anemia increases the risk of all-cause mortality [23], as low levels of hemoglobin (the carrier of oxygen from blood to cells) results in hypoxia, which causes multiple cell dysfunctions and eventually respiratory distress [24]. Data about the association between dyslipidemia and death upon COVID-19 is relatively limited and conflicting, however, dyslipidemia was found to be a contributing risk factor for developing cardiovascular diseases, hence, increases the severity of COVID-19 [25]. Li *et al*. *(2020)* reported that COVID-19 and progression of the disease cause rapid alteration in the lipid profile and several abnormalities [26]. Another study by Feingold *et al*. (2020) showed that HDL levels were significantly lower in severe cases of COVID-19 [27]. In contrast, Wei *et al*. (2020) study found that hypolipidemia was associated with higher risk of developing severe symptoms of COVID-19 [22]. However, the most likely explanation that supports our finding is that human cell membrane is composed of cholesterol, which controls the fusion proteins of toxins or viruses and increases their infectivity by altering and modulating the dynamics of the membranes. Thus, high levels of human cholesterol in the membranes promotes the fusion of the SARS-CoV-2 peptides, enhances its entry to cells and influences life cycle of the virion as assembling and budding [28, 29]. Moreover, the prevalence of chronic kidney disease among SARS-CoV-2 hospitalized patients resulted in higher risk of in-hospital mortality [30]. A study conducted in Wuhan, China showed a remarkable escalation of serum creatinine among 101 COVID-19 hospitalized patients, which resulted in 33.7% in-hospital mortality [30]. Thereby, it is crucial to assess the chronic kidney disease or developed acute renal injury in the hospital to identify patients at higher risk of severe COVID-19 manifestations.

Since the UAE is a multi-national and globalized environment with different cultural integration, it was important to highlight this diversity in our study to investigate the impact of different ethnicities on the mortality following COVID-19 among UAE populations [31]. The present study revealed that patients from Middle eastern origin were at higher risk of death compared to other nationalities living in the UAE. The other nationalities were derived mainly from Asia. The significantly high death rate among the Middle eastern can be attributed to several justifications, the first is related to the “hygiene hypothesis”, which has been evolved in the 90s and correlates between the environmental experience with microbes and immunity system status. The hypothesis advocated that exposure to environmental pollutants (farms, parasites, and pets) at young age were less likely to acquire infections and autoimmune diseases compared to populations living in more hygienic circumstances. The frequent use of antimicrobial agents as hand sanitizers and soaps contributed to commensal microorganisms imbalance in the body systems, thereby, weakening the immunity system to fight other microorganisms including SARS Cov-2 [32, 33]. Asians’ population are usually living in crowded environments which may contribute to hygiene theory. The second possible justification is the high prevalence of obesity, dyslipidemia, anemia, type 2 diabetes and its complications like kidney diseases among Middle eastern population, which are significantly associated with more severe and lethal illness of COVID-19 [34, 35]. In the current study for example the average BMI for patients of middle eastern origin was significantly higher than those of other origins (30.3 vs 26.9, p = 0.001). The third possible justification is that Asians living in UAE are usually workers of younger age than emirates and other middle eastern populations. This is true for the study sample as the average age of Middle Eastern patients was 49.4 as compared to 42.9 for other patients. Furthermore, another justification of this finding could be attributed to the human leukocyte antigen (HLA), where HLA alleles varies significantly within populations, hence, patients may develop different immune response and pathophysiological changes to infectious diseases [36]. Novelli et al analyzed the HLA allele frequency distribution in a group of 99 Italian patients affected by the severe form of COVID‐19 and found a significant association when compared to a control group for three alleles: HLA B*27:07 (0.10% vs 2:02%, p = 0.004), DRB1*15:01 (4.62% vs 10.10%, p = 0.0480), DQB1*06:02 (3.64% vs 7.58%, p = 0.016) [37]. This indicates the possible impact of specific HLA alleles on COVID-19 severity and outcomes.

According to our findings, the most common clinical manifestations of COVID-19 were cough and fever. This is consistent with previously published study conducted study in China [30]. However, in some cases with low immunity, COVID-19 may infest with normal body temperature. Shortness of breath/Dyspnea is another common symptom of COVID-19 as it reflects lung dysfunction due to low level of oxygen without fever [18]. Therefore, when patient is complaining from difficulty of breathing without fever, further assessment of other deterioration should be addressed to identify the health problem. Furthermore, some patients may suffer from an olfactory dysfunction during COVID-19, which is sensory disorder involving loss of smell and/or taste even after complete clearance of the infection from the body [38]. In contrast, this study showed only 1.1% of the patients having olfactory dysfunction. This could be due to that most patients in this study suffered from mild disease. The occurrence of olfactory dysfunction is highly variable among the COVID-19 patients ranging from 5.0 to 98.0% depending on the region and demographic characteristics of the patients. In addition, olfactory function is a transient symptom that mainly occurs within the first 7 days of the infection among patients in the emergency rooms and ICU compared to outpatient settings. Remission of olfactory function may delay even after recovery and viral clearance, the possible explanation is that the neuron progenitor cells could be infected by SARS-COV-2 and impair the generation of olfactory sensory neuron [39, 40].

During this study, most of the hospitalized cases were mild and few of them were transferred to the ICU (4.3%). Although few cases were transferred to the ICU, the risk of death was high almost double the risk among non-ICU cases. This can be clearly justified by the respiratory distress affecting COVID-19 patients, which requires invasive mechanical ventilation in critical cases and ends with a definite death [41]. On the other hand, a previously published meta-analysis reported a pooled rate of ICU admission (10.9%), higher than the reported rate of this study [42] and another study in Italy presented even higher rate of ICU admission (87.3%) among 3988 hospitalized COVID-19 patients that was associated with an absolute death [43].

## Strengths and limitations

The strength of this study is that it included a large sample size of 3296 patients, which is a representative number of UAE population and allows generalizability of the findings.

Despite the large number of COVID-19 patients in the hospital presented in this study, timeframe and data extraction were limited to 3 months and conducted in a single site. Also, due to the relatively small number of mortality cases, other identified risk factors from univariate analysis such as age, respiratory rate and VitD deficiency should also be taken into consideration.

## Conclusion

The results indicated that most COVID-19 cases were mild and morality rate was low compared to worldwide reported mortality. Mortality rate during hospitalization was higher in patients from Middle East origin with preexisting comorbidities especially anemia, COPD, and chronic kidney disease. Due to the relatively small number of mortality cases, other identified risk factors from univariate analysis such as age, respiratory rate and VitD deficiency should also be taken into consideration. It is crucial to stratify patients on admission based on these risk factors to help decide regarding intensity and type of treatment. Further studies are required to investigate the treatment approaches for reducing the risk of death of COVID-19.

## Supporting information

S1 Data Study data. (SAV)
